# The rice *BRITTLE CULM 4* gene encodes a membrane protein affecting cellulose synthesis in the secondary cell wall

**DOI:** 10.1093/pcp/pcaf096

**Published:** 2025-08-21

**Authors:** Masatoshi Yamaguchi, Ami Sato, Daisuke Takahashi, Kazuhisa Mori, Ryota Fujimoto, Atsuko Miyagi, Eriko Sato, Toshiki Ishikawa, Ryosuke Sano, Tetsuya Kurata, Shiro Suzuki, Yasuko Kaneko, Maki Kawai-Yamada, Toshihisa Kotake

**Affiliations:** Graduate School of Science and Engineering, Saitama University, 255 Shimo-Okubo, Sakura-ku, Saitama 338-8570, Japan; Graduate School of Science and Engineering, Saitama University, 255 Shimo-Okubo, Sakura-ku, Saitama 338-8570, Japan; Graduate School of Science and Engineering, Saitama University, 255 Shimo-Okubo, Sakura-ku, Saitama 338-8570, Japan; Graduate School of Science and Engineering, Saitama University, 255 Shimo-Okubo, Sakura-ku, Saitama 338-8570, Japan; Graduate School of Science and Engineering, Saitama University, 255 Shimo-Okubo, Sakura-ku, Saitama 338-8570, Japan; Graduate School of Science and Engineering, Saitama University, 255 Shimo-Okubo, Sakura-ku, Saitama 338-8570, Japan; Faculty of Agriculture, Yamagata University, 1-23 Wakaba-machi, Tsuruoka, Yamagata 997-8555, Japan; Graduate School of Science and Engineering, Saitama University, 255 Shimo-Okubo, Sakura-ku, Saitama 338-8570, Japan; Graduate School of Science and Engineering, Saitama University, 255 Shimo-Okubo, Sakura-ku, Saitama 338-8570, Japan; Graduate School of Science and Technology, Nara Institute of Science and Technology, 8916-5 Takayama-cho, Ikoma, Nara 630-0192, Japan; Graduate School of Science and Technology, Nara Institute of Science and Technology, 8916-5 Takayama-cho, Ikoma, Nara 630-0192, Japan; Integrated Initiative for Designing Future Society, Kyushu University, Fukuoka, 819-0395 Japan; Faculty of Applied Biological Sciences, Graduate School of Natural Science and Technology, and the United Graduate School of Agricultural Science, Gifu University, 1-1 Yamagido, Gifu 501-1193, Japan; Graduate School of Science and Engineering, Saitama University, 255 Shimo-Okubo, Sakura-ku, Saitama 338-8570, Japan; Faculty of Education, Saitama University, 255 Shimo-Okubo, Sakura-ku, Saitama 338-8570, Japan; Graduate School of Science and Engineering, Saitama University, 255 Shimo-Okubo, Sakura-ku, Saitama 338-8570, Japan; Graduate School of Science and Engineering, Saitama University, 255 Shimo-Okubo, Sakura-ku, Saitama 338-8570, Japan; Green Bioscience Research Center, Saitama University, 255 Shimo-Okubo, Sakura-ku, Saitama 338-8570, Japan

**Keywords:** brittle culm, cellulose synthesis, genome editing, membrane protein, rice (*Oryza sativa*), secondary cell wall

## Abstract

The formation of secondary cell walls, which provide mechanical strength to the plant body, depends on numerous factors. Studies on rice *brittle culm* (*bc*) mutants allow us to identify these factors and gain insights into the mechanisms of secondary cell wall formation. Rice *bc4* is a recessive *bc* mutant with fragile culms and leaves, similar to other *bc* mutants. We found that the *bc4* mutant exhibited reduced cellulose content in the culm cell walls compared to the japonica cultivar Taichung 65 and the indica cultivar Kasalath, while hemicellulose content remained unchanged. Transmission electron microscopy revealed reduced cell wall thickness in the sclerenchyma cells of the *bc4* culm, indicating that BC4 contributes to normal cellulose synthesis or deposition in secondary cell walls. Positional cloning and subsequent genome sequencing revealed that the *BC4* gene encodes a four α-helical transmembrane protein with 205 amino acids, and that the *bc4* mutation results in a premature termination codon in this gene. Four *bc4* mutants generated from the japonica cultivar Nipponbare, using genome editing with the CRISPR/Cas9 system, exhibited reduced cellulose content along with *bc* phenotypes. Gene clustering analysis based on expression patterns and metabolomic analysis suggested that BC4 functions independently from secondary cell wall cellulose synthase catalytic subunits and COBRA-like protein. These results suggest that the BC4 protein is a newly identified factor involved in cellulose synthesis or deposition in the secondary cell walls of rice.

## Introduction

Secondary cell walls, generally rich in cellulose, xylan, and lignin, are deposited inside primary cell walls after cell growth and furnish the plant body with mechanical strength. As the synthesis and deposition of these cell wall components are developmentally controlled and require the interplay of many factors cooperating in various roles, the mechanism for secondary cell wall formation is still elusive.

To date, many genes necessary for secondary cell wall formation have been identified in studies on Arabidopsis (*Arabidopsis thaliana*) cell wall mutants. Arabidopsis *irregular xylem* (*irx*) mutants show collapsed morphology in xylem tissues that normally have thickened cell walls. The *irx1*, *irx3*, and *irx5* mutants appear to have defects in secondary cell wall-specific cellulose synthase catalytic subunits (CesAs) (Turner and Somerville 1997, Taylor et al. 1999, 2000, 2003). Glycosyltransferase genes involved in the synthesis of secondary cell wall xylan have also been cloned from Arabidopsis *irx7*, *irx8*, *irx9*, *irx10*, and *irx14* mutants (Brown et al. 2007, Peña et al. 2007, Persson et al. 2007, Wu et al. 2009). On the other hand, a series of studies on Arabidopsis *fragile fiber* (*fra*) mutants with reduced mechanical strength of the inflorescence stem has identified other factors including katanin, kinesin-like protein, and phosphatidylinositol phosphatase, together with CesAs, as factors necessary for normal secondary cell wall formation (Burk et al. 2001, Zhong et al. 2002, 2003, 2004, Zhang et al. 2009).

Along with mutant analysis, candidate factors important for cellulose synthesis and deposition in primary and secondary cell walls have been identified through co-expression analysis (Brown et al. 2005, Persson et al. 2005). Companions of cellulose synthase proteins (At5g42860 and At1g45688), highly co-expressed with primary cell wall CesAs, have been shown to interact with CesAs and contribute to microtubule organization and localization of the cellulose synthase complex (CSC) on the plasma membrane in Arabidopsis (Endler et al. 2015). TONNEAU1 (At2g45900), highly co-expressed with a secondary cell wall *CesA* gene, *CesA7/IRX5*, was later shown to be a microtubule-associated protein that localizes to cortical microtubules in Arabidopsis (Drevensek et al. 2012). On the other hand, a membrane protein (At4g27435, AtUNKA) belonging to the domain with unknown function (DUF) 1218 family has not yet been characterized, although this gene is also co-expressed with secondary cell wall-specific CesA genes (Ubeda-Tomas et al. 2007). Other DUF1218 proteins, MODIFYING WALL LIGNIN 1 (MWL1, At1g31720) and MWL2 (At4g19370), are presumed to have roles in secondary cell wall formation, based on the observation that an Arabidopsis *mwl1 mwl2* double mutant exhibited slightly reduced lignin content in the inflorescence stem (Mewalal et al. 2016).

The *brittle culm* (*bc*) mutants of *Poaceae* plants exhibit reduced mechanical strength, especially in culms and leaves (Kokubo et al. 1989, 1991, Ching et al. 2006, Sindhu et al. 2007). Because many mutants in this class are morphologically normal, they have been expected to have defects in the secondary cell walls rather than the primary cell walls. The *brittle stalk* (*bs*), *culm easily fragile* (*cef*), and *fragile culm* (*fc*) mutants of Poaceae plants also show *bc* phenotypes, and it has been demonstrated that they have defects in secondary cell walls as well (Li et al. 2003, Ching et al. 2006, Ye et al. 2015, Zhang et al. 2020). At least ten *bc*, two *cef*, and three *fc* mutants have been characterized so far in rice (Li et al. 2003, Yan et al. 2007, Zhou et al. 2009, Hirano et al. 2010, Kotake et al. 2011, Zhang et al. 2012, Wu et al. 2012, Sakai et al. 2013, Ye et al. 2015, Zhang et al. 2020, Ma et al. 2021, Li et al. 2022, Jiang et al. 2022, Ruan et al. 2022, Dang et al. 2023) (Supplementary Table S1). Among these mutants, a decrease in the cellulose content is commonly observed, suggesting that cellulose synthesis and deposition in the secondary cell wall are central events for the mechanical strength of the plant body. This is supported by the fact that secondary cell wall-specific CesAs have been identified as causal genes for several *bc* mutants. On the other hand, the proper formation of secondary cell walls requires many factors other than CesAs. Interestingly, the factors identified through the analysis of rice mutants are not necessarily the same as those in Arabidopsis. Rice *bc* mutants thus remain interesting as materials for gaining new insights into the mechanisms of secondary cell wall formation.

Rice *bc4* is one of the *bc* mutants yet to be studied (Khush et al. 1984, Khush 1995, Librojo and Khush 1986). In the present study, we characterized *bc4* mutants through cell wall fractionation followed by sugar composition analysis and transmission electron microscopy (TEM) observation. The *BC4* gene has been mapped to chromosome 6 on the classical linkage map. Through positional cloning and genome sequencing, we identified a premature stop mutation in a gene encoding a membrane protein with four α-helices. Based on the clustering of expression patterns, we suggest that the *BC4* gene contributes to normal cellulose synthesis and/or deposition through an unknown function, distinct from those of the *CesA* and *COBRA-like* genes.

## Results

### Reduced cellulose content in the *bc4* mutant

**Figure 1:**
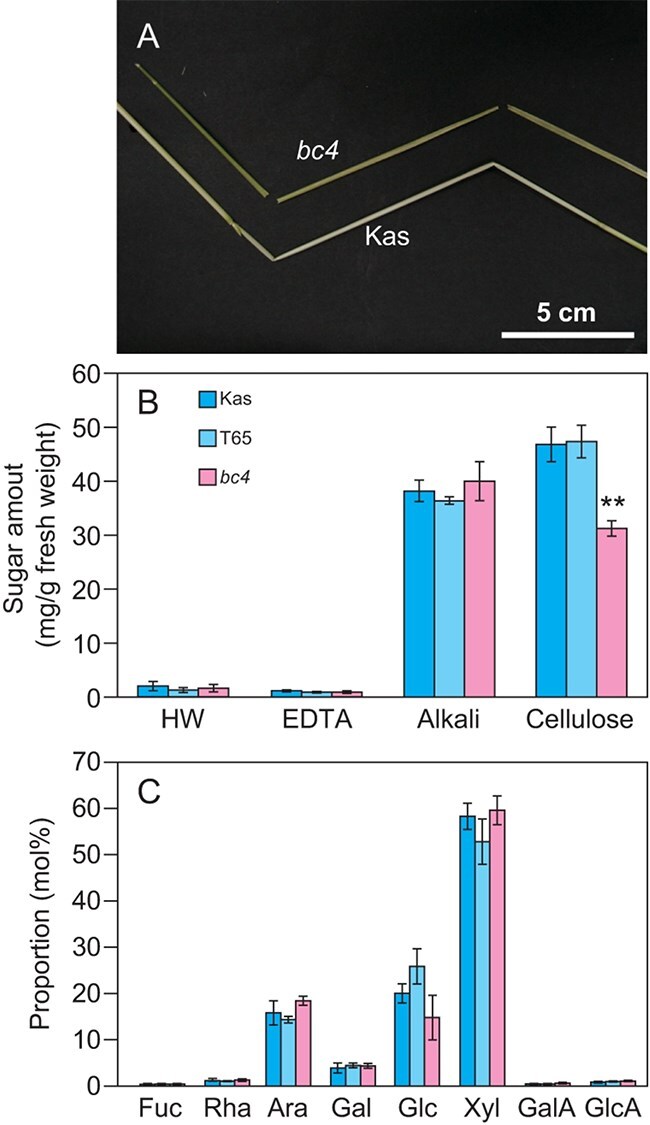
The *bc* phenotype and reduced cellulose content. (A) the *bc* phenotype observed in the uppermost culm. (B) Amount of cell wall fractions. Sugar amounts of cell wall fractions extracted from culms were measured two weeks after heading. The proportions of fractions in total cell wall sugar are shown in Supplementary Fig. S1. (C) Sugar composition of alkali fraction. The sugar composition of the alkali fraction was determined by HPAEC-PAD. The left and central bars, and the right bars indicate data for WT T65, Kas, and the *bc4* mutant, respectively. Data are mean values with SD (*n* = 4 biological replicates). The asterisk indicates significant difference from Kas plants (Student’s *t* test, ^**^, *P* < 0.01).

The rice *bc4* mutant has been known for >40 years, but has remained uncharacterized (Khush et al. 1984, Librojo and Khush 1986). The *bc4* mutant exhibited a weak dwarf phenotype compared to other indica cultivars such as Kasalath (Kas) (data not shown). Similar to other *bc* mutants, the plant body of the *bc4* mutant lacks mechanical strength, which is presumed to be caused by defects in the secondary cell walls (Fig. 1A). However, the *bc* phenotypes of *bc4* mutants are sometimes less pronounced compared to other *bc* mutants such as *bc1* and *Bc6*, suggesting that the functions of BC4 are different from those of COBRA-like and CesA proteins, which are impaired in rice *bc1* and *Bc6* mutants, respectively (Li et al. 2003, Sato et al. 2010, Kotake et al. 2011).

To address the changes in secondary cell walls in the *bc4* mutant, we first fractionated cell wall polysaccharides of the culm into a hot water (HW) fraction, an ethylenediaminetetraacetic acid (EDTA) fraction containing pectin, an alkali fraction mainly containing arabinoxylan, and a cellulose fraction by sequential extraction and compared the content of these fractions with an indica cultivar, Kas, and a japonica cultivar, Taichung 65 (T65). We used these two cultivars for comparison because we could not obtain the normal line with the same genetic background as *bc4*. Consistent with our previous studies on rice cell walls (Aohara et al. 2009, Hirano et al. 2010, Kotake et al. 2011), the culms mainly consisted of alkali and cellulose fractions. The *bc4* mutant had a significantly lower cellulose fraction content than T65 and Kas (Fig. 1B, Supplementary Fig. S1). On the other hand, the content and sugar composition of the alkali fraction rich in arabinoxylan were comparable to those in normal cultivars (Fig. 1C), indicating that only cellulose content was changed in the culm of the *bc4* mutant.

### Tissue morphology and cell wall thickness of sclerenchyma cells

**Figure 2:**
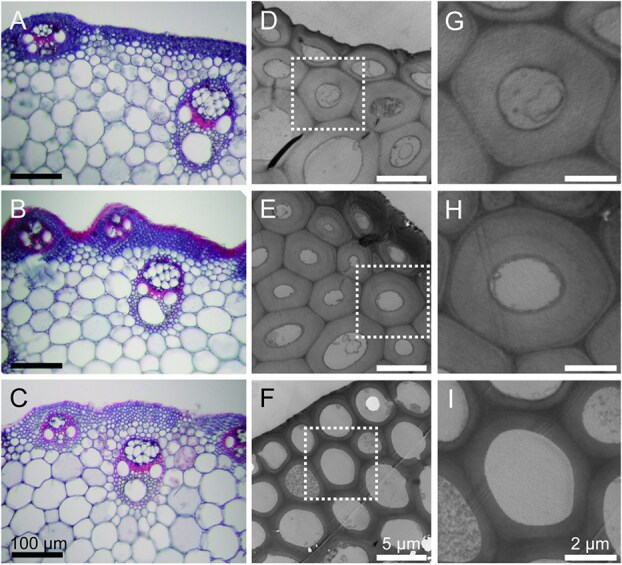
Tissue morphology and cell walls in *bc4* culms. Transverse sections of uppermost culms of T65 (A), Kas (B), and *bc4* (C) were stained with safranin O and fast Green FCF. Sclerenchyma cell walls of T65 (D, G) and Kas (E, H) were compared with the *bc4* culm (F, I) cell wall by TEM observation. The boxed areas in D-F are magnified in G-I. Cell wall thickness measurements are in Supplementary Fig. S3.

In Arabidopsis, defects in secondary cell walls result in altered tissue organization, such as collapsed xylem (Turner and Somerville 1997, Taylor et al. 2000, 2003). To address the influence of the *bc4* mutation on tissue morphology, microscopic observation of transverse sections of *bc4* culms stained with Safranin O and Fast Green FCF was conducted. The *bc4* mutant did not show any collapsed xylem phenotype at all (Fig. 2A-C). However, tissue staining with Safranin O showed weaker red coloration in the *bc4* mutant compared to T65 and Kas. Since Safranin O is known to stain lignified cell walls (Bond et al. 2008), transverse sections were treated with phloroglucinol to selectively stain lignin-rich cell walls. However, we did not observe a clear difference in *bc4* from Kas and T65 in phloroglucinol staining (Supplementary Fig. S2A-C). Quantitative analysis using the Klason method did not reveal a significant difference in lignin content (Kirk and Obst 1988) (Supplementary Fig. S2D).

To address the changes in cell walls in the *bc4* mutant, TEM of culm transverse sections was also conducted. Although we did not observe obvious changes in tissue morphology, the cell wall thickness of sclerenchyma cells of the *bc4* culm decreased compared with T65 and Kas (Fig. 2D-I, Supplementary Fig. S3). These results suggest that the *bc4* mutant is impaired in cellulose synthesis or deposition in the culm.

**Figure 3:**
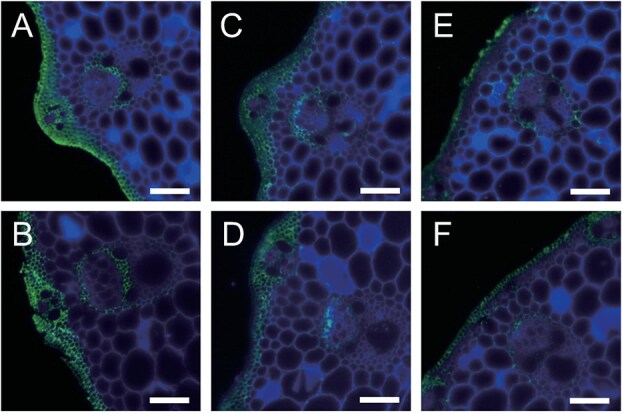
Accumulation of xylan in *bc4* culms. Xylan in the cross sections from the top internode of NP (A, C) and *bc4* mutant (B, D) was detected and compared with LM10 specific to heteroxylan and LM28 specific to glucuronoxylan. For comparison, AGP was also detected with LM2 specific to D-glucuronosyl residues of AGP in the NP (E) and the *bc4* mutant (F). Merged images of Alexa Fluor 488 and Calcofluor white are shown. The bars indicate 50 μm.

Arabinoxylan deposition in the *bc4* mutant was also compared by immunostaining with LM10 and LM28 specific to xylan with another normal japonica cultivar, Nipponbare (NP). Xylan deposition in sclerenchyma and vascular cells was similar in the culms of the *bc4* mutant and NP (Fig. 3).

### Cloning of the *bc4* gene

**Figure 4:**
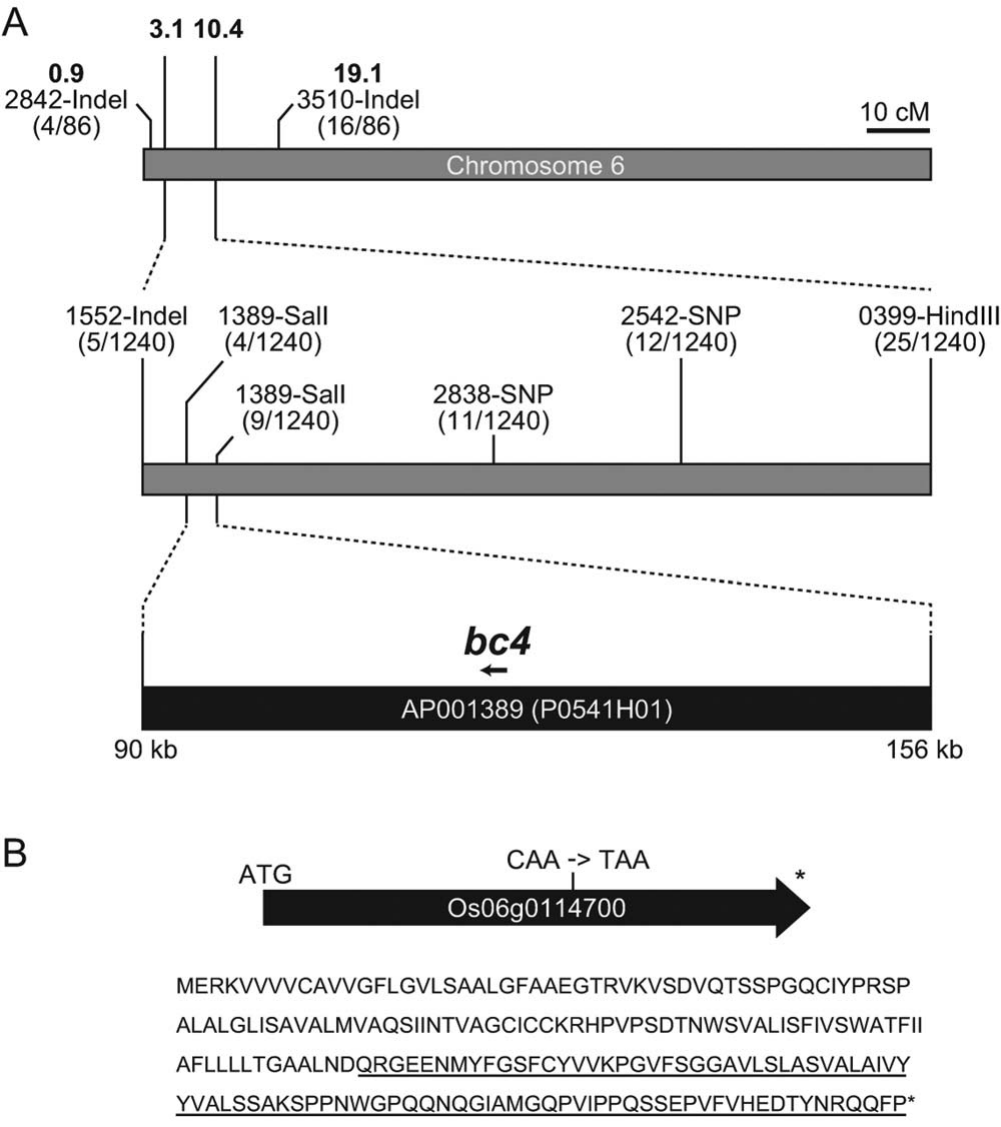
Cloning of *BC4*. (A) Positional cloning of the *BC4* gene. The *bc4* locus in chromosome 6 was confirmed by rough mapping using an F2 population generated by crossing *bc4* mutant and co39, and then with 620 F2 plants delimited to a 66 kb region in a BAC clone, AP001389 (P0541H01). The numbers of recombinants among the F2 individuals analyzed are shown. The primers for DNA markers are listed in Supplementary Table S2. (B) the *bc4* mutation in ORF Os06g0114700. Genome sequencing identified a mutation in Os06g0114700 resulting in a premature stop codon, deleting a C-terminal region of 87 amino acids (underlined).

The *bc4* locus was previously mapped to chromosome 3 in the rice classical linkage map through trisomic tests (Librojo and Khush 1986), but the locus has been changed to chromosome 6 (https://shigen.nig.ac.jp/rice/oryzabase/marker/mapCirn/6). We first performed rough mapping of the *bc4* gene using the F2 population generated by crossing the *bc4* mutant with an indica cultivar, co39. We confirmed that the *bc4* gene locates between the 2842-Indel and 3510-Indel DNA markers in chromosome 6 (Fig. 4A). In the following fine mapping with six DNA markers designed between these two markers, the *bc4* locus was narrowed down to a 66 kb region corresponding to the bacterial artificial chromosome (BAC) clone AP001389 (P1-derived artificial chromosome, P0541H01) in chromosome 6 (Fig. 4A).

To identify the mutation that causes the *bc* phenotype, we obtained the whole genome sequence of the *bc4* mutant by next-generation sequencing. By comparison of the genome sequence in the delimited 66 kb region between the *bc4* mutant and NP, we found one point mutation from C to T, affecting molecular function, in an open reading frame, Os06g0114700 (Fig. 4B). Other than this mutation, there was no mutation that changed amino acid sequences in this region. This gene encoded a four α-helical transmembrane protein with 205 amino acids belonging to the DUF1218 family (Krogh et al. 2001) (Supplementary Fig. S4), and the mutation resulted in a premature stop codon deleting the C-terminal region of 87 amino acids (Fig. 4B). In Arabidopsis, a DUF 1218 gene (At4g27435) is co-expressed with secondary cell wall CesA genes and two DUF1218 genes (At1g31720 and At4g19370), which have been shown to be involved in the lignin deposition by analysis of the double mutant (Brown et al. 2005, Persson et al. 2005, Mewalal et al. 2016), although the amino acid sequences of these Arabidopsis proteins were not highly related to Os06g0114700. This is the first time that a loss-of-function mutation of DUF1218 has been characterized in Poaceae plants.

### *bc4* mutants generated by genome editing

**Figure 5:**
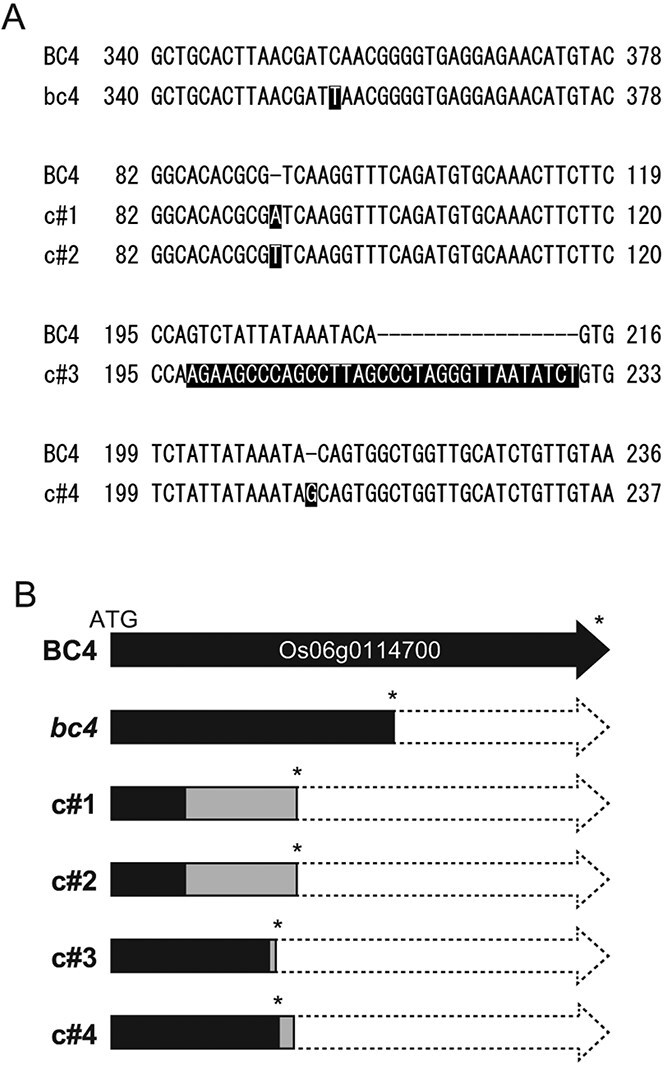
The *bc4* mutants generated by genome editing. (A) Mutations introduced in the mutant lines c#1–4. The genomic sequences of c#1–4 were aligned and compared with the NP sequence. The nucleotides of c#1–4 differing from NP are highlighted in black. The premature stop codons are underlined. (B) Schematic diagram of gene products from the *bc4* mutant and lines c#1–4. The region identical to NP is shown in black, and that changed from NP due to the frame shift or the insertion of unknown fragments is shown in gray.

In order to confirm that the *bc4* mutation is a loss-of-function mutation in Os06g0114700, other alleles of *bc4* mutants were generated from the japonica cultivar NP by genome editing using CRISPR-Cas9 technology (Mikami et al. 2015) (Fig. 5A). We obtained four independent mutant lines, c#1 to 4, with different mutations. In the c#1, 2, and 4 lines, base-insertion resulted in a frame-shift and a premature stop codon, while in the c#3 line, the targeted region was replaced with an unknown sequence including a stop codon in the frame. These genome-edited (GE) lines all encoded proteins much shorter than Os06g0114700 (Fig. 5B).

**Figure 6:**
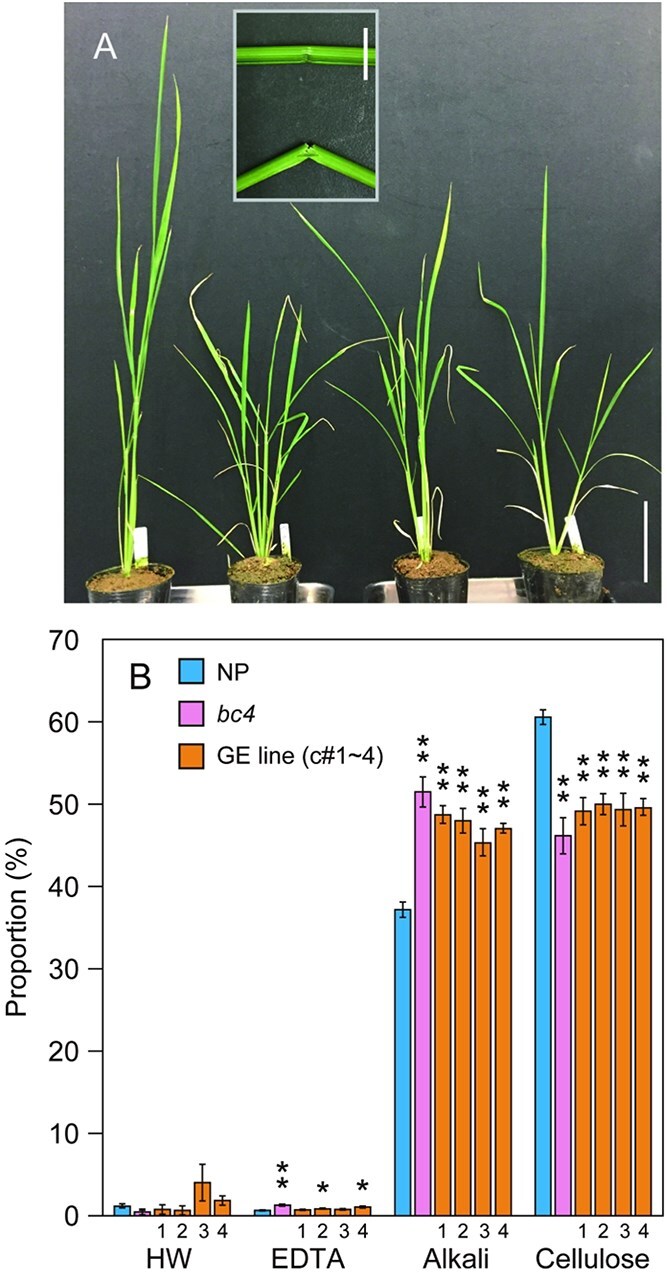
Cell wall properties of GE lines. (A) Growth of the GE lines. From left to right, 52-day-old specimens of NP, *bc4*, c#3, and c#4. Bar = 10 cm. The fragile phenotype of GE line c#3 is shown in the inset (bar = 1 cm). (B) Cell wall fractions of lines c#1–4 compared to *bc4* and NP. As in the results shown in Fig. 1, the cell walls of line c#1–4 culms were fractionated and quantified. Note that the proportions of fractions are shown here because fresh weight differed in biological replicates. Data are mean values with SD (*n* = 3 biological replicates). GE, genome-edited line. The asterisk indicates a significant difference from the NP plant (Student’s *t* test, ^*^, *P* < 0.05, ^**^, *P* < 0.01).

Consistent with the phenotype of *bc4* mutant, GE lines c#1 to 4 had *bc* phenotypes and reduced cellulose content in cell walls (Fig. 6). In addition, semi-dwarf phenotypes were also observed in c#3 and 4 (Supplementary Fig. S5). These results confirmed that the gene encoding a DUF1218 protein is the causal gene of the rice *bc4* mutant.

## Discussion

### Reduced cellulose content in the *bc4* mutant

Cellulose synthesis and deposition are essential processes that provide mechanical strength to the rice plant body. Consistent with other *bc* mutants, the *bc4* mutant showed a significantly lower cellulose content in the culm compared to T65 and Kas, a finding also confirmed in GE lines of NP (Fig. 6; Supplementary Fig. S1). However, the effects on cell wall properties aside from cellulose are not identical across rice *bc* mutants. For instance, in the *bc3* mutant, the alkali fraction, which mainly contains arabinoxylan, remained unchanged (Hirano et al. 2010), whereas in the *Bc6* mutant, this fraction significantly increased, likely as a compensatory response (Kotake et al. 2011). Although we could not perform a detailed cell wall analysis on the GE lines of NP, *bc4* did not show a significant difference in the sugar composition of the alkali fraction or in lignin content compared with the japonica cultivar T65, which likely shares a highly related genetic background with NP. (Ichida et al. 2023, Guo et al. 2025). These results suggest that the impact of the *bc4* mutation on other cell wall components differs from that of the *Bc6* mutation in the *CesA9* gene (Fig. 1).

### Functions of *BC4* in secondary cell wall formation

To gain insights into *BC4* function, we performed hierarchical clustering analysis based on expression patterns with other *bc* genes (Brown et al. 2005, Persson et al. 2005, Miki et al. 2019). The *bc* genes clustered into at least three distinct groups. One group included cellulose synthesis-related genes, such as *BC1* (*COBRA-like 4*), *BC6* (*CesA9*), *BC7/BC19* (*CesA4*), and *CEF1* (*Myb103-like*), while another clade included genes involved in xylan synthesis, represented by *FC18* (*UDP-Xyl synthase*) and *FC19* (*IRX10*) (Li et al. 2003, Roudier et al. 2005, Liu et al. 2013, Ruan et al. 2022, Dang et al. 2023) (Supplementary Fig. S6). *BC4* clustered within a membrane trafficking/cytoskeleton-related group, alongside *BC3* (*dynamin-related protein DRP2B*), *BC12* (*kinesin-4*), and *CEF3* (*membrane-trafficking protein SCD2*). The co-expressed genes were also confirmed in the Rice FREND database (https://ricefrend.dna.affrc.go.jp/) (Sato et al. 2013). The secondary cell wall *CesA* genes were not included in a list of genes co-expressed with *BC4*, whereas *BC6* is co-expressed with *BC1* (*COBRA-like 4*) and *OsCesA7*, which encodes another secondary cell wall CesA (Supplementary Figs S7 and S8). These findings suggest that *BC4* may contribute to secondary cell wall formation in conjunction with membrane trafficking genes, such as *BC3*, *BC12*, and *CEF3* (Hirano et al. 2010, Zhang et al. 2012, Jiang et al. 2022).

Since secondary cell wall formation requires large amounts of nucleotide sugars such as UDP-D-glucose (UDP-Glc) and UDP-D-xylose (UDP-Xyl), disruptions in cell wall formation in *bc* mutants may be related to altered sugar metabolism. In our previous study, we compared the metabolites in the *bc4* mutant with those in other *bc* mutants (Miyagi et al. 2022); however, due to differences in genetic backgrounds, clear distinctions in the *bc4* mutant from the japonica cultivar T65 or indica cultivar IR68 were not observed. In this study, we identified metabolites that significantly increased or decreased in the culms of c#2 compared to NP (Supplementary Fig. S9). Notably, levels of UDP-D-glucuronic acid (UDP-GlcA)/UDP-D-galacturonic acid (UDP-GalA) were significantly elevated. Although not statistically significant, UDP-Glc/UDP-D-galactose (UDP-Gal) also tended to increase in c#2. It is probable that impaired cellulose synthesis increased the levels of UDP-Glc and UDP-GlcA, which is formed from UDP-Glc in the UDP-sugar pathway (Kotake et al. 2016). On the other hand, these shifts in UDP-sugars contrast with patterns in the *bc1* and the *Bc6* mutants, suggesting that the *BC4* gene is involved in an unknown process distinct from cellulose synthesis (Miyagi et al. 2022).

### Relationship of the BC4 protein with other DUF1218 proteins

Amino acid sequences with high similarity to BC4 are found in various land plants, including lycophytes and mosses, suggesting that this type of protein is widely distributed among plants. However, based on phylogenetic analysis (Supplementary Fig. S10), closely related homologs of BC4 are not highly conserved in land plants. In addition, in Arabidopsis, no cell wall defects have been reported in the loss-of-function mutant of BC4’s closest homolog (At5G17210). These facts suggest that DUF1218 proteins are redundant or not important for secondary cell wall formation in Arabidopsis.

The 3D structure of the plant DUF1218 protein has not yet been characterized experimentally. The 3D structure predictions generated using AlphaFold2 (Google Colab) and the Phyre2 server suggest that the BC4 protein shares structural similarity with mammalian claudin proteins, which are also transmembrane proteins composed of four α-helices. (Supplementary Fig. S3B) (Kelley et al. 2015, Jumper et al. 2021, Mirdita et al. 2022). Mammalian claudin proteins are known to function in tight junctions, acting as impermeable barriers for fluid on the plasma membrane (Nguyen et al. 2024). However, because plants have cell walls, it is unlikely that BC4 would seal intercellular spaces in the same manner. In addition, the different topological arrangement (clockwise versus anticlockwise) of α-helices and the loss of a relatively large domain between first and second α-helices suggest that BC4 protein functions differently from mammalian claudin.

The intracellular localization of the BC4 protein was predicted using the WoLF PSORT program, with comparisons to other proteins that share features with BC4 (Horton et al. 2007). Among the 14 proteins listed for BC4, nine are predicted to localize to the vacuole. By contrast, the closest homolog of BC4 in Arabidopsis (At5G17210) is a plasma membrane protein. While it remains possible that BC4 has a specific role in cellulose synthesis, it is more likely that BC4 serves a general function, such as membrane protein transport or the regulation of plasma membrane properties. Further studies are needed to clarify the molecular functions of BC4.

## Materials and Methods

### Plant materials

Seeds of *bc4* mutant (RGS number 293) and co39 were provided by the International Rice Research Institute (IRRI, Los Baños, Laguna, Philippines) (Librojo and Khush 1986, Khush 1995). Seeds of T65 and Kas were obtained from the National Genetic Institute (Mishima, Shizuoka, Japan) and Genebank of the National Institute of Agrobiological Sciences (NIAS, Tsukuba, Ibaraki, Japan), respectively. T65 and Kas were mainly used to represent wild-type (WT) controls. Rice plants were grown under field conditions at NIAS or indoors at 28°C under a 12 h light/12 h dark photoperiod condition.

### Analysis of cell wall polysaccharides

Cell wall polysaccharides were fractionated into HW, EDTA, alkali, and cellulose fractions as described (Kotake et al. 2011). Plant tissues were first crushed to powder using a mortar and pestle in liquid nitrogen and then homogenized in water. The homogenates were washed twice with water, suspended in 80% (v/v) ethanol, and heated at 100°C for 15 min, and then treated with 100 units of α-amylase from porcine pancreas (Sigma-Aldrich, St. Louis MO, USA) in 50 mM sodium 3-morpholinopropanesulfonic acid buffer (pH 6.5) at 37°C for 4 h. Released starch was removed by centrifugation at 1500 *g*. Cell wall polysaccharides were sequentially extracted at 100°C for 10 min with water thrice, 50 mM EDTA (pH 6.8) thrice, and 17.5% (w/v) NaOH containing 0.04% (w/v) NaBH_4_ thrice. The residual precipitate was washed with water, ethanol, and diethyl ether, and collected as the cellulose fraction. Hemicellulose was neutralized with acetic acid, dialyzed against water at 4°C for 1 day, and lyophilized. The cellulose fraction was hydrolyzed with 72% (v/v) sulfuric acid at 25°C for 1 h, followed by 8% (v/v) sulfuric acid at 100°C for 4 h (Sawake et al. 2015). The sugar content in each fraction was determined by the phenol-sulfuric acid method (Dubois et al. 1956) using Glc as the standard.

### Determination of sugar composition

The dialyzed alkali fraction was hydrolyzed with 72% (v/v) sulfuric acid (0.2 ml) at 25°C and then 8% (v/v) sulfuric acid at 100°C as described above. The hydrolysate was neutralized with barium carbonate and desalted with Dowex 50 W (H^+^) resin. The content of each monosaccharide was determined by high-performance anion-exchange chromatography with pulsed amperometric detection (HPAEC-PAD) using a Dionex DX-500 liquid chromatograph equipped with a CarboPac PA-1 column and a pulsed amperometric detector (Thermo Fisher Scientific, Waltham, MA, USA) as described previously (Ishikawa et al. 2000, Sawake et al. 2015). In this study, the proportion of L-arabinose (Ara), L-fucose (Fuc), Gal, GalA, Glc, GlcA, D-mannose (Man), L-rhamnose (Rha), and Xyl was determined.

### Histology

For the observation of cross sections, the uppermost internode was used. Fixation, dehydration, embedding, and sectioning were performed as described (Kotake et al. 2011). The sections (thickness, 20 μm) were stained with 0.1% (w/v) Safranin O (Nacalai Tesque, Kyoto, Japan) for 24 h, washed in a series of ethanol/water solutions (50%–95% ethanol, v/v), stained with 0.05% (w/v) Fast Green FCF (Wako, Tokyo, Japan) for 45 s, and washed with ethanol. The sections were observed under a light microscope (Eclipse E400, Nikon, Tokyo, Japan).

The xylan and arabinogalactan-protein (AGP) in the culms of *bc4* mutant and NP plants grown indoors were observed. Sections excised from the uppermost internode were fixed, dehydrated through a graded ethanol series, and embedded in Technovit 7100 (Nisshin EM, Tokyo, Japan) using a graded series of ethanol and Technovit 7100 mixtures. Then, the sections were solidified with Technovit hardener (Nisshin EM) at 60°C for 12 h. Sections were sliced to a thickness of 5 μm using a microtome (RM2125RT; Leica, Tokyo, Japan) equipped with a disposable steel blade (C35; Feather, Osaka, Japan).

The acetyl groups of glucomannan and xylan were removed in advance by treatment with 50 mM sodium hydroxide at 25°C for 30 min. The sections were washed with phosphate-buffered saline (PBS) containing 137 mM NaCl, 10 mM disodium hydrogenphosphate, 2.7 mM KCl, and 1.8 mM potassium dihydrogenphosphate (pH 7.4) and blocked in PBS containing 0.3% (w/v) blocking milk (Sigma-Aldrich, Tokyo, Japan) for 30 min. Then, the sections were incubated for 1.5 h with antibodies against AGP (LM2, Kerafast, Shirley, MA; dilution 1:5) or xylan (LM10, Kerafast; dilution 1:5; LM28, Kerafast; dilution 1:5) in PBS with blocking milk. After washing three times with PBS, the sections were incubated for 1 h with Alexa Fluor 488-conjugated anti-mouse IgM (Abcam, Cambridge, UK; dilution 1:100) for LM2 and LM28 or Alexa Fluor 488-conjugated anti-mouse IgG (Abcam; dilution 1:100) for LM10 in PBS with blocking milk. After washing three times with PBS, the cross sections were also stained with 0.025%(w/v) Calcofluor White in PBS for 5 min. The cross sections were imaged with a microscope (FV1000D, Olympus, Tokyo, Japan) at 405 nm for Calcofluor White and at 488 nm for Alexa Fluor 488.

### Transmission electron microscopy

Observation of sclerenchyma cells in culms by TEM was performed as described previously (Hirano et al. 2010, Kotake et al. 2011). Briefly, pieces excised from uppermost culms (1–2 mm) were fixed in 2% (v/v) glutaraldehyde in 50 mM potassium phosphate buffer (pH 7.0) at room temperature for 2 h and then at 4°C overnight. After rinsing with the same buffer, tissues were postfixed in 2% (w/v) OsO_4_ in the buffer. After dehydration in a graded series of acetone, the tissues were embedded in Spurr’s resin, which was polymerized by incubation at 70°C overnight. Ultra-thin sections of 90–100 nm thickness were cut with a diamond knife and stained with 2% (w/v) uranyl acetate for 15 min, followed by lead citrate for 2 min. The sections were observed with a Hitachi H-7500 electron microscope (Hitachi Science Systems, Ibaraki, Japan) at an accelerating voltage of 100 kV.

### Positional cloning

For positional cloning of the *BC4* gene, *bc4* was crossed with the indica variety co39. The resulting F2 population was analyzed for *bc* phenotype and genotype. For the rough mapping of *BC4*, the genotypes of 43 plants were analyzed with Indel DNA markers, 2842-Indel and 3510-Indel (Supplementary Table S2). Then fine-mapping was performed by analyzing the genotypes of 620 plants using Indel, cleaved amplified polymorphic sequence, and SNP DNA markers, 1552-Indel, 1389-SalI, 1389-SNP, 2838-SNP, 2542-SNP, and 0399-HindIII (Supplementary Table S2). These DNA markers were generated by direct sequencing of 1–2 kb PCR DNA fragments amplified by genomic PCR of *bc4* mutant and co39.

### Genome sequencing

Genome DNA of the *bc4* mutant was extracted with a FavorPrep Plant Genome DNA Extraction Mini Kit (Favorgen, https://www.favorgen.com/en/product.php). The library for genome sequencing was prepared according to Uchida et al. (2011). The library was sequenced by Illumina Genome Analyzer IIx (Illumina, https://illumina.com). Obtained single end reads (ca. 140 million, 75 bp) were mapped onto Os-Nipponbare-Reference-IRGSP-1.0 (https://rapdb.dna.affrc.go.jp), and effective SNPs on gene function were analyzed by using Strand NGS (Strand Life Sciences, USA) according to Yamauchi et al. (2016).

### Generation of *bc4* mutants by genome editing

To obtain the double-strand target sequence, oligo DNA fragments (for c#1 and c#2; 5′- GTTGGGCGGAGGGCACACGCGTCA-3′ and 5’-AAACTGACGCGTGTGCCCTCCGCC-3′, and for c#3 and c#4; 5’-GTTGCCAGTCTATTATAAATACAG-3′ and 5’-AAACCTGTATTTATAATAGACTGG-3′) were incubated at 95°C for 5 min, and then at room temperature for 20 min to be annealed. The double-strand target sequence was ligated to pU6gRNA-oligo (Mikami et al. 2015) linearized with BbsI. The obtained plasmids, which contained the guide RNA driven by OsU6 promoter, were digested with PacI and AscI, and ligated with PacI and AscI sites of pZH_OsU3gYSA_MMCas9 vector (Mikami et al. 2015). The resultant vectors were used for generation of transgenic rice (NP) plants according to Toki et al. (2006). The resulting *bc4* mutants c#1–4 were grown indoors.

### Metabolomic analysis

The internodes (2 weeks after heading) of *bc4* mutant c#4 and NP grown indoors were used. The metabolomic analysis was performed as described previously (Miyagi et al. 2010, 2019, 2022). Briefly, metabolites were extracted with 50% methanol containing 50 μM piperazine-1,4-bis(2-ethanesulfonic acid) and 50 μM methionine sulfone as internal standards and measured by capillary electrophoresis-triple quadrupole mass spectrometry (CE, 7100; MS, G6420A; Agilent Technologies, Santa Clara, CA, USA) or liquid chromatography-triple quadrupole-mass spectrometry (LC-MS-8030, Shimadzu GLC, Kyoto, Japan) with multi-reaction monitoring.

## Supplementary Material

pcp-2024-e-00271-File009_pcaf096

pcp-2024-e-00271-File008_pcaf096

## Data Availability

The data for genome sequencing have been deposited in DDBJ [https://ddbj.nig.ac.jp/search/entry/bioproject/PRJDB19637] with accession number PRJDB19637.
