# Supplementary material for: The rice BRITTLE CULM 4 gene encodes a membrane protein affecting cellulose synthesis in the secondary cell wall
Source: Plant Cell Physiol. 2025 Aug 21;66(10):1444–53. doi: 10.1093/pcp/pcaf096 (PMC12540249; doi:10.1093/pcp/pcaf096)
Supplement: pcp-2024-e-00271-File009_pcaf096 [file pcp-2024-e-00271-file009_pcaf096.pdf]

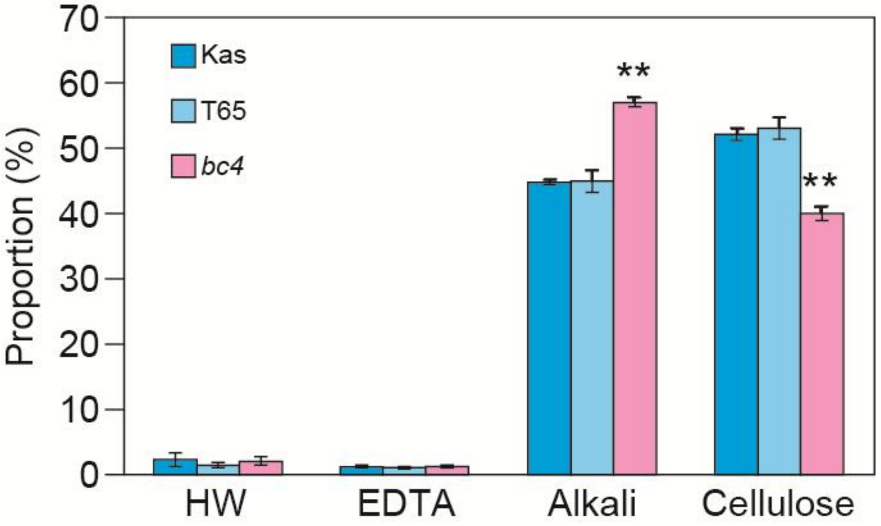


**Supplementary Figure 1. Proportion of cell wall fractions in *bc4* mutant.** The proportions of fractions based on the sugar amount were calculated from the data shown in Fig. 1B. The asterisk indicates a significant difference from NP (Student's t test, **, P < 0.01).

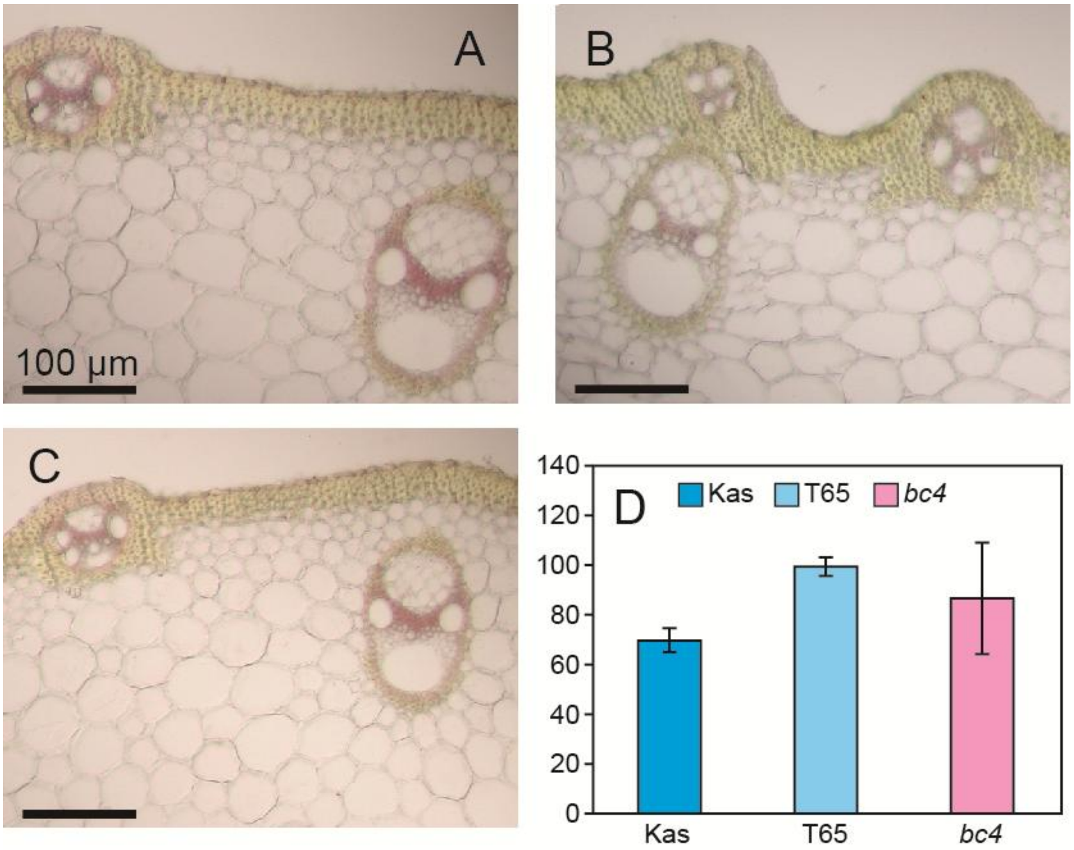


**Supplementary Figure 2. Lignin accumulation in *bc4* mutant.** To detect lignin in cell walls, culm cross-sections (thickness, 30 μm) of Kas (A), T65 (B), and *bc4* (C) were washed with ethanol to remove paraffin, stained with 2% (w/v) phloroglucinol (Wako, Tokyo, Japan) and then treated with 18% (w/v) HCl. (D)  Klason lignin was quantified (Kirk and Obst, 1988). No significant decrease in lignin accumulation was observed in the culm of *bc4* mutant plants.

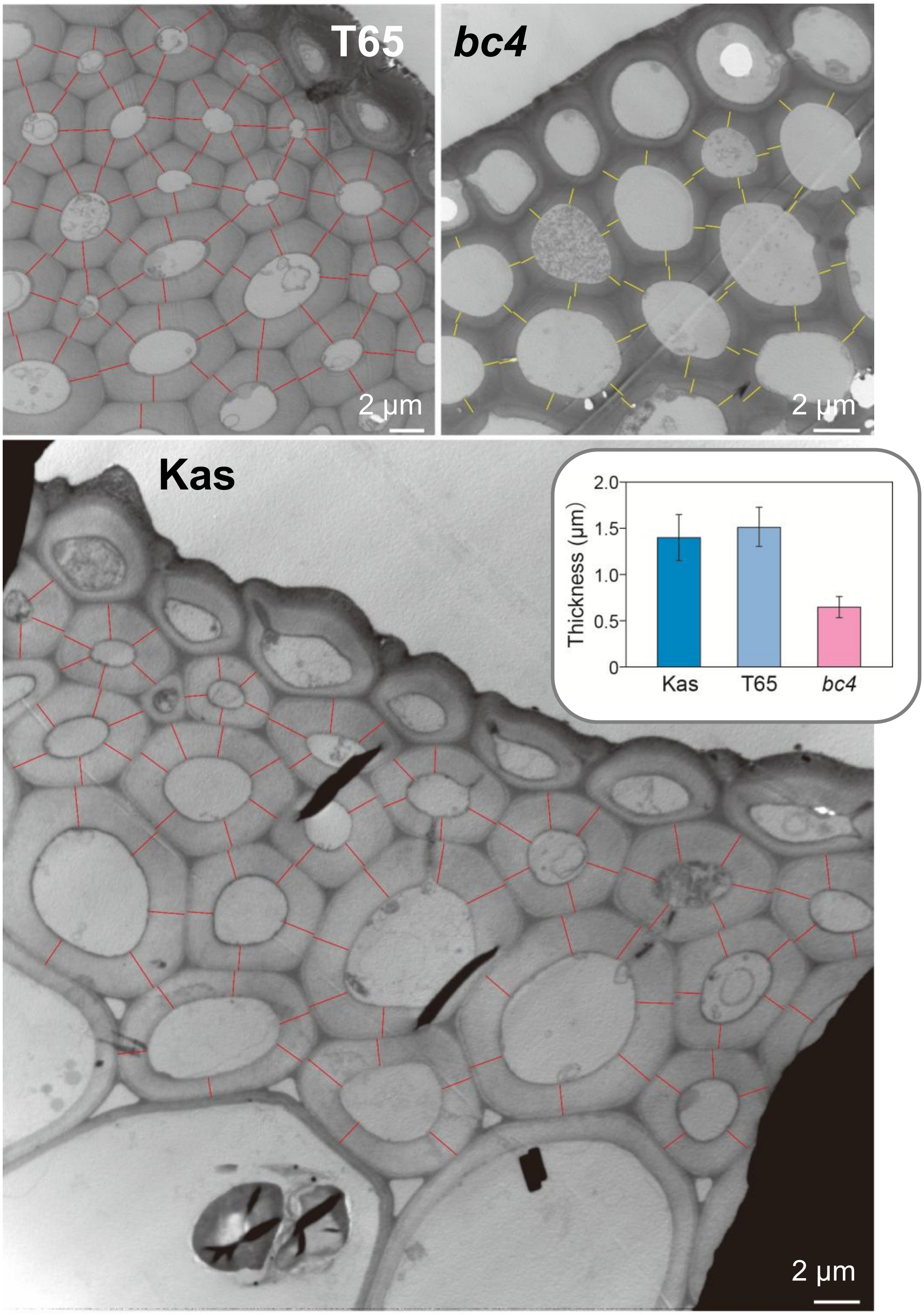


**Supplementary Figure 3. Reduced cell wall thickness in the culm of *bc4* mutant.** The cell wall thickness of sclerenchyma cells of the top culm 2 weeks after heading was measured in pictures shown here. The *bc4* mutant had thinner cell walls (0.65 ± 0.12 µm) than Kas (1.41 ± 0.25 µm), and T65 (1.52 ± 0.21 µm). The inset graph shows the average thickness of T65 (112 technical replicates), *bc4* mutant (57 technical replicates), and Kas (100 technical replicates). The thicknesses measured are shown in red (T65 and Kas) and yellow (*bc4*) bars.

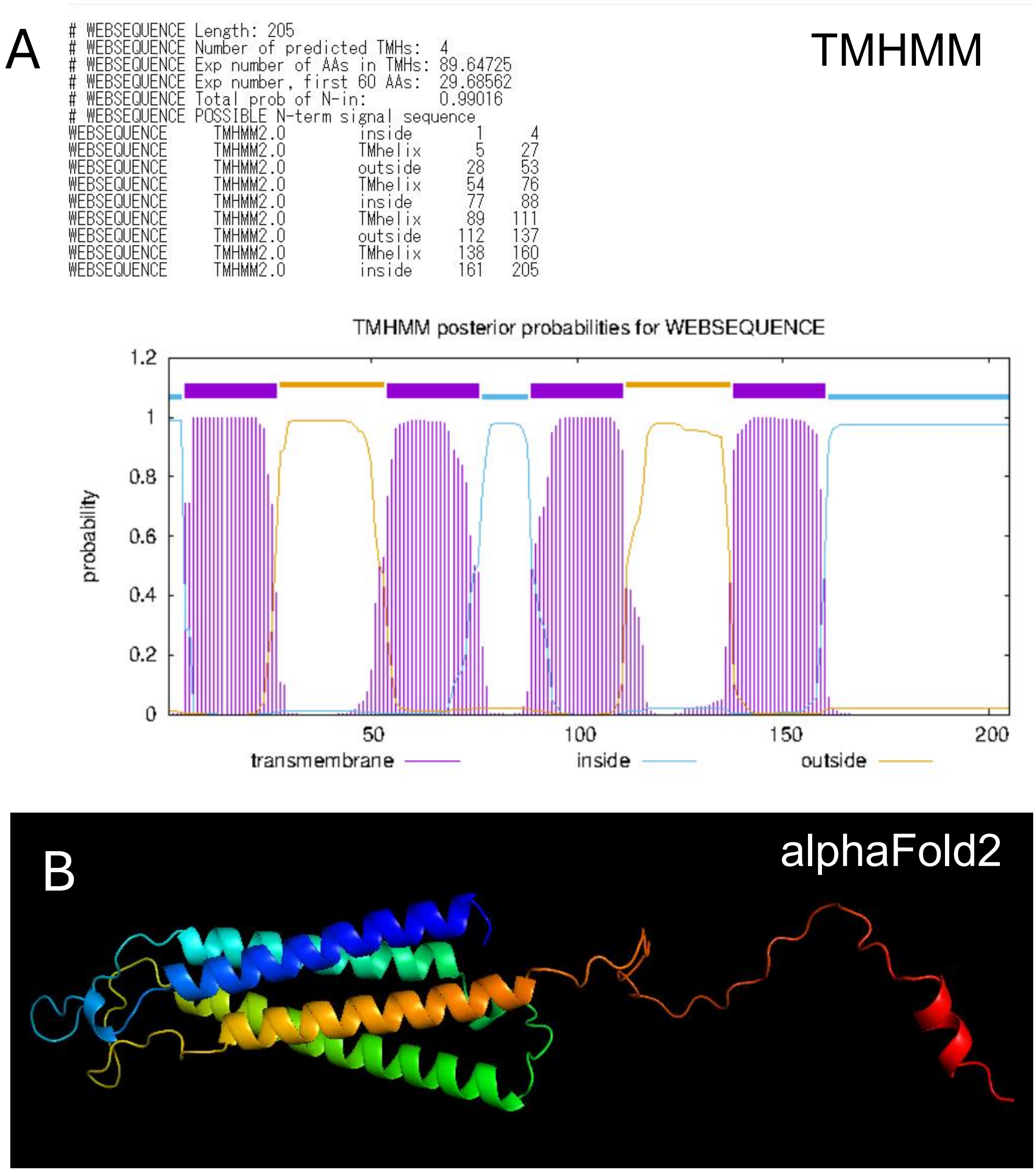


**Supplementary Figure 4. The four-α-helical transmembrane protein encoded by the *BC4* gene.** (A) The transmembrane regions were predicted by the TMHMM program (Krogh et al, 2001). (B) The 3D structure was predicted by alphaFold2 (Jumper et al., 2018).

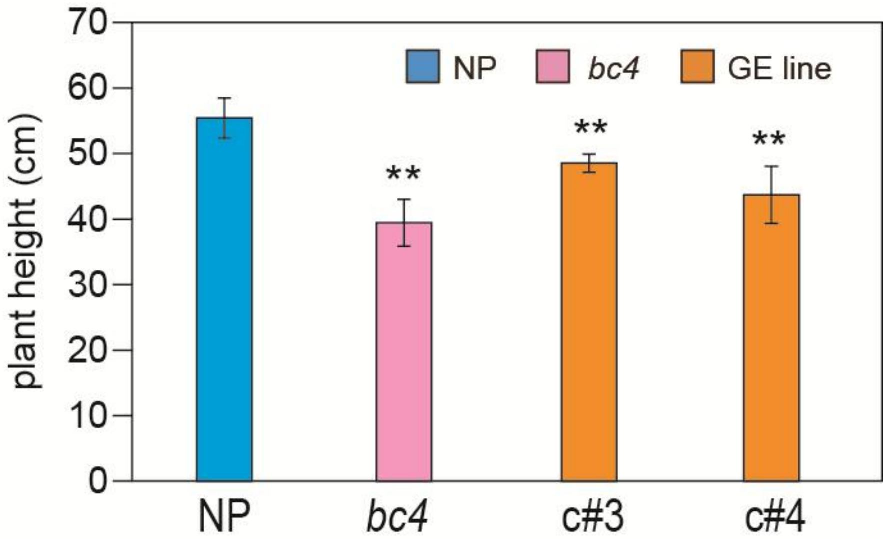


**Supplementary Figure 5. Reduced plant height caused by *bc4* mutation.** Plant height after heading was compared. The double asterisk indicates significant a difference from NP (n =5, Student's *t* test, $P < 0.01$).

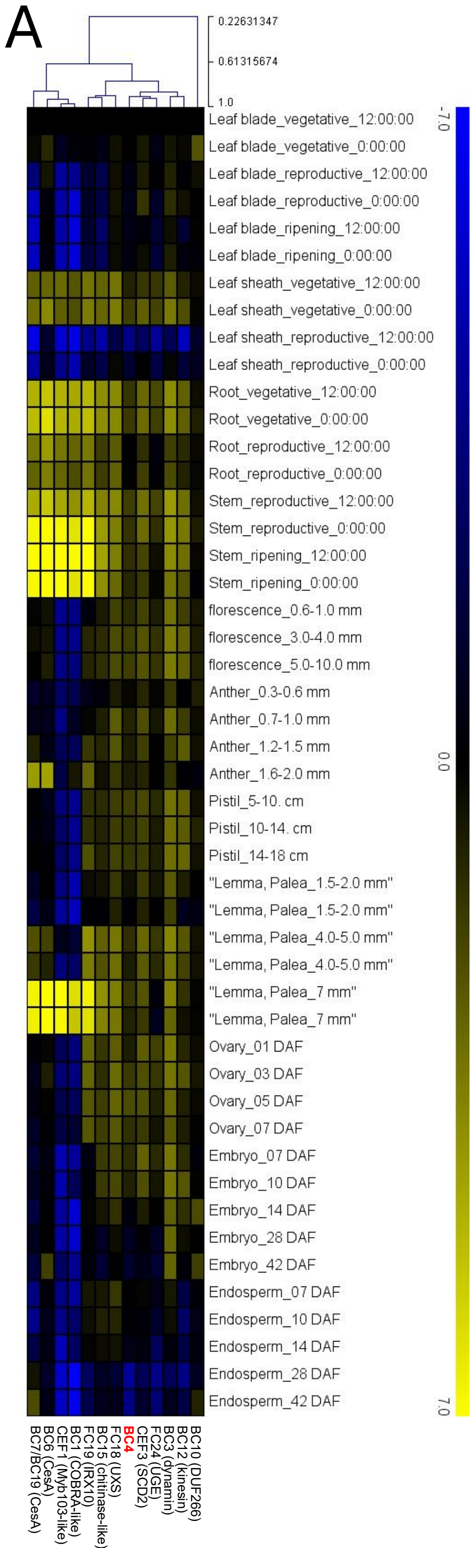


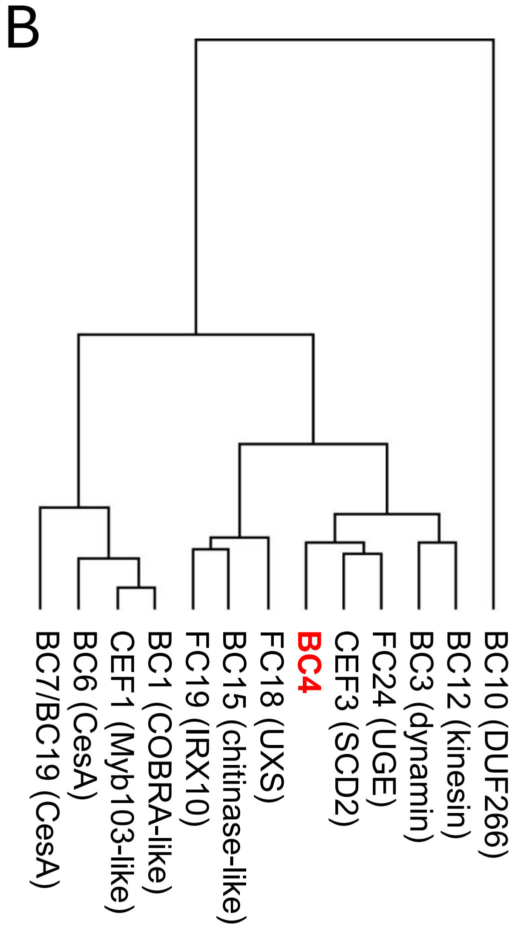


**Supplementary Figure 6. Gene clustering based on expression patterns.** (A) Gene expression data for *BC4*, *BC1*, *BC3*, *BC6*, *BC7/BC19*, *BC10*, *BC12*, *BC15*, *BC17*, *CEF1*, *CEF3*, *FC18*, *FC19*, and *FC24* were collected from Rice Annotation Project Database (RAP-DB, https://rapdb.dna.affrc.go.jp/) (Li et al. 2003; Hirano et al. 2010; Kotake et al. 2011; Yan et al. 2007; Ma et al. 2021; Zhou et al. 2009 Zhang et al. 2012; Wu et al. 2012: Li et al. 2022; Ye et al. 2015; Jiang et al. 2022; Ruan et al. 2002; Dang et al. 2023; Zhang et al. 2020; Sakai et al. 2013). Gene clustering was conducted in accordance with a previous study (Miki et al., 2019) with slight modification. Briefly, hierarchical clustering was performed by Multi-Experiment Viewer (MeV ver. 4.9) using log2-transformed fold change values relative to Leaf_blade_vegetative_12:00:00. (B) The tree obtained in the analysis is magnified.

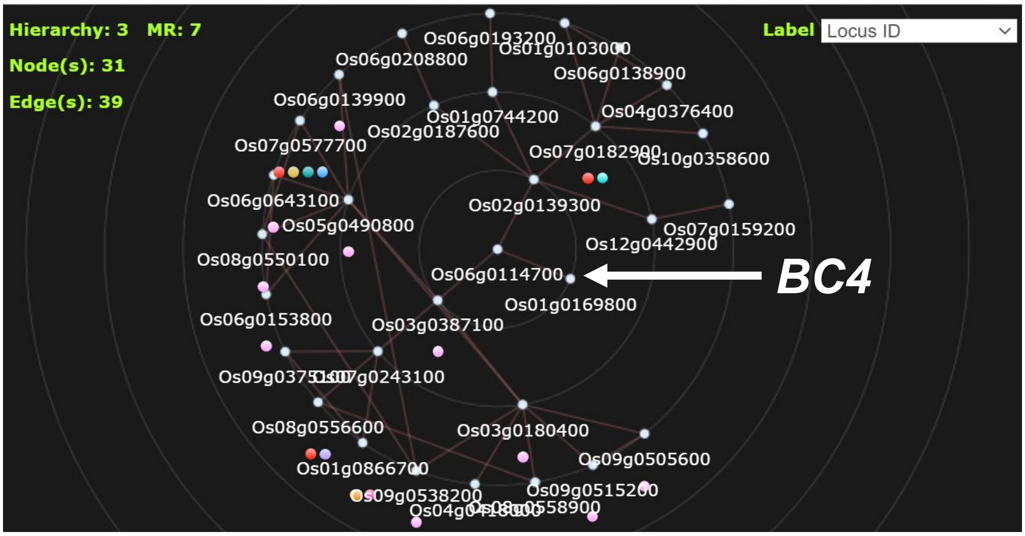


| Locus ID | Annotation | LOC ID |
|---|---|---|
| Os01g0169800 | Allinase, C-terminal domain containing protein. | LOC_Os01g07500 |
| Os02g0139300 | Glycoside hydrolase, family 17 protein. | LOC_Os02g04670 |
| Os03g0387100 | Proteasome subunit alpha type 2 (EC 3.4.25.1) (20S proteasome alpha subunit B) (20S proteasome subunit alpha-2). | LOC_Os03g26970 |
| Os01g0744200 | Conserved hypothetical protein. | LOC_Os01g54090 |
| Os02g0187600 | Conserved hypothetical protein. | LOC_Os02g09470 |
| Os03g0180400 | Proteasome subunit alpha type 6 (EC 3.4.25.1) (20S proteasome alpha subunit A) (20S proteasome subunit alpha-1). | LOC_Os03g08280 |
| Os05g0490800 | Similar to Proteasome subunit alpha type 3 (EC 3.4.25.1) (20S proteasome alpha subunit G). | LOC_Os05g41180 |
| Os07g0182900 | Similar to Cytosine-5 DNA methyltransferase MET1 (Fragment). | LOC_Os07g08500 |
| Os07g0243100 | Similar to Mitochondrial import inner membrane translocase subunit Tim10. | LOC_Os07g13950 |
| Os12g0442900 | DNA-binding SAP domain containing protein. | LOC_Os12g25640 |
| Os01g0103000 | Non-protein coding transcript, unclassifiable transcript. | (not found) |
| Os01g0866700 | Similar to Sm-like protein. | LOC_Os01g64690 |
| Os04g0376400 | Glycoside hydrolase, family 18 protein. | LOC_Os04g30770 |
| Os04g0418000 | Conserved hypothetical protein (SDH5). | LOC_Os04g34100 |
| Os06g0138900 | Conserved hypothetical protein. | LOC_Os06g04699 |
| Os06g0139900 | Similar to Beta 1 subunit of 20S proteasome. | LOC_Os06g04800 |
| Os06g0153800 | Beta 5 subunit of 20S proteasome. | LOC_Os06g06030 |
| Os06g0193200 | Similar to Pectin methylesterase 8 (Fragment). | LOC_Os06g09340 |
| Os06g0208800 | Hypothetical protein. | LOC_Os06g10660 |
| Os06g0643100 | Proteasome subunit beta type 3 (EC 3.4.25.1) (20S proteasome alpha subunit C) (20S proteasome subunit beta-3). | LOC_Os06g43570 |
| Os07g0159200 | Non-protein coding transcript, uncharacterized transcript. | LOC_Os07g06530 |
| Os07g0577700 | ATP-citrate lyase/succinyl-CoA ligase domain containing protein. | LOC_Os07g38970 |
| Os08g0550100 | Similar to 26S proteasome subunit RPN3a. | LOC_Os08g43640 |
| Os08g0556600 | Conserved hypothetical protein. | LOC_Os08g44250 |
| Os08g0558900 | Similar to F1F0-ATPase inhibitor protein. | LOC_Os08g44460 |
| Os09g0375100 | Non-protein coding transcript, uncharacterized transcript. | LOC_Os09g20830 |
| Os09g0505600 | Proteasome subunit beta type 1 (EC 3.4.25.1) (20S proteasome alpha subunit F) (20S proteasome subunit beta-6). | LOC_Os09g32800 |
| Os09g0515200 | Beta 7 subunit of 20S proteasome. | LOC_Os09g33986 |
| Os09g0538200 | Proteasome subunit alpha type 7 (EC 3.4.25.1) (20S proteasome alpha subunit D) (20S proteasome subunit alpha-4). | LOC_Os09g36710 |
| Os10g0358600 | Conserved hypothetical protein. | LOC_Os10g21460 |

**Supplementary Figure S7.** Genes co-expressed with *BC4*. A hyper tree and the gene list are shown. These data were acquired from Rice FREND (https://ricefrend.dna.affrc.go.jp/gene-search.php?keyword=Os06g0114700).

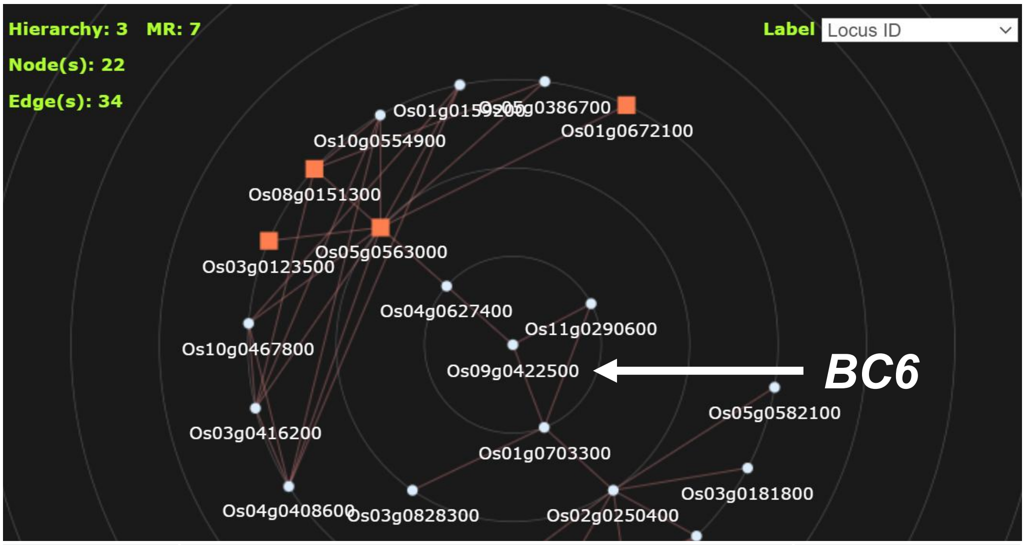


| Locus ID | Annotation | LOC ID |
|---|---|---|
| Os01g0703300 | Zinc finger, RING-type domain containing protein. | LOC_Os01g50750 |
| Os04g0627400 | PAK-box/P21-Rho-binding domain containing protein. | LOC_Os04g53580 |
| Os11g0290600 | Similar to Serpin. | LOC_Os11g18660 |
| Os02g0250400 | Similar to GDSL-motif lipase/hydrolase-like protein. | LOC_Os02g15230 |
| Os03g0828300 | Similar to (1-4)-beta-mannan endohydrolase-like protein. | LOC_Os03g61270 |
| Os05g0563000 | No apical meristem (NAM) protein domain containing protein (NAC). | LOC_Os05g48850 |
| Os01g0159200 | Conserved hypothetical protein. | LOC_Os01g06580 |
| Os01g0672100 | No apical meristem (NAM) protein domain containing protein (NAC). | LOC_Os01g48130 |
| Os01g0675500 | Similar to Glycoprotein-specific UDP-glucuronyltransferase-like protein. | LOC_Os01g48440 |
| Os02g0329800 | Protein of unknown function DUF563 family protein. | LOC_Os02g22380 |
| Os03g0123500 | Similar to HOS66 protein (HB). | LOC_Os03g03164 |
| Os03g0181800 | Protein of unknown function DUF936, plant family protein. | LOC_Os03g08390 |
| Os03g0416200 | **BRITTLE CULM1 (BC1).** | LOC_Os03g30250 |
| Os04g0408600 | Protein of unknown function DUF662 family protein. | LOC_Os04g33450 |
| Os05g0386700 | Conserved hypothetical protein. | (not found) |
| Os05g0582100 | Cas1p-like family protein. | (not found) |
| Os06g0595800 | Transferase family protein. | LOC_Os06g39470 |
| Os06g0687900 | Glycosyl transferase, family 43 protein. | LOC_Os06g47340 |
| Os08g0151300 | Myb, DNA-binding domain containing protein. | LOC_Os08g05520 |
| Os10g0467800 | **Similar to Cellulose synthase (Fragment, CesA7).** | LOC_Os10g32980 |
| Os10g0554900 | Protein of unknown function DUF566 family protein. | LOC_Os10g40620 |

**Supplementary Figure S8.** List of genes co-expressed with *BC6*. A hyper tree and the gene list are shown. These data were acquired from Rice FREND (https://ricefrend.dna.affrc.go.jp/gene-search.php?keyword=Os09g0422500).

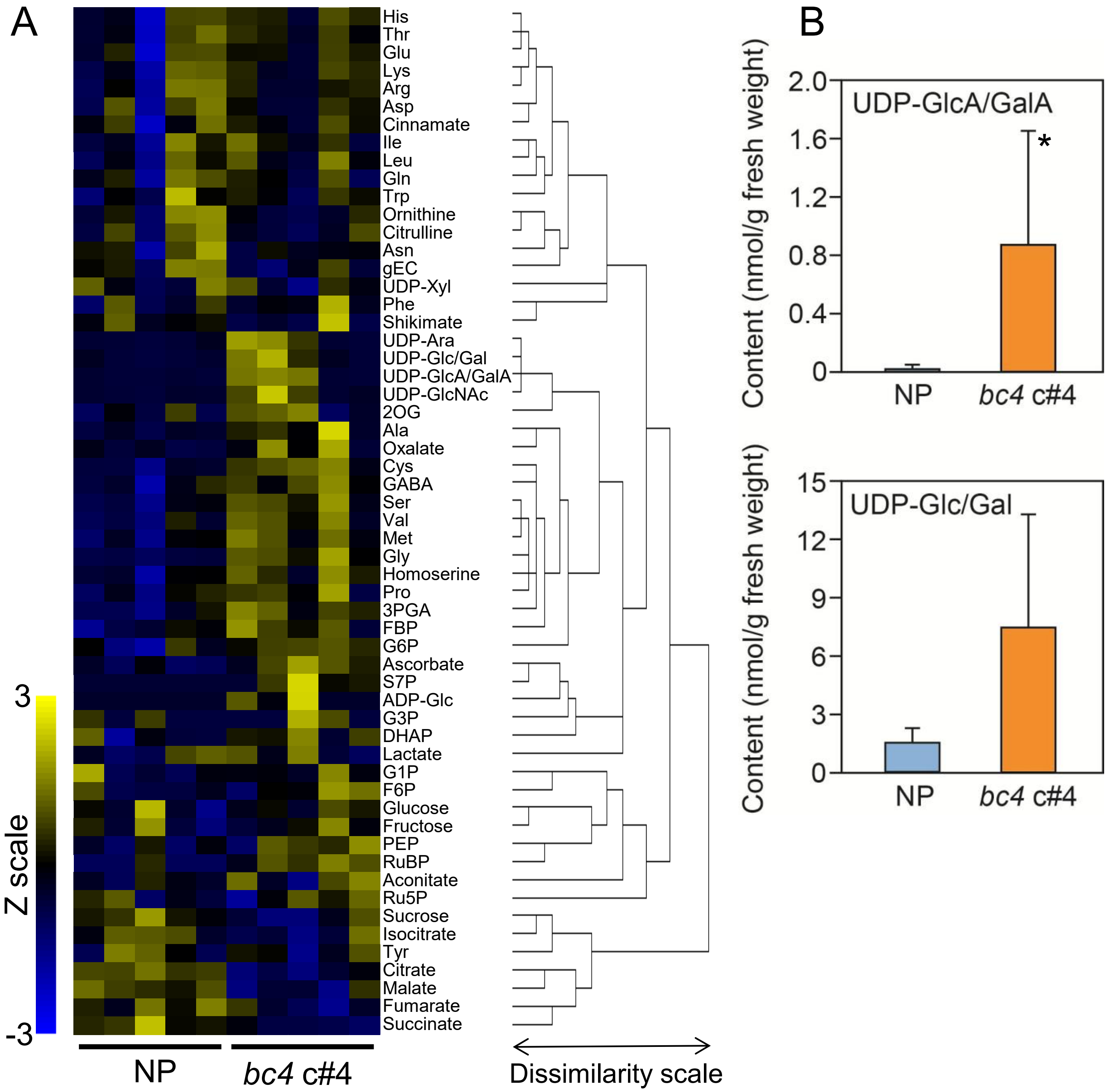


**Supplementary Figure 9. Metabolic changes in internodes of *bc4* mutant.** (A) A heatmap with a metabolite dendrogram obtained by hierarchical clustering analysis. The top internodes 2 weeks after heading of *bc4* mutant c#2 and NP were analyzed. (B) Contents of UDP-sugars. The asterisk indicates significant difference from NP (n =5, Student's *t* test, $P < 0.05$). Together with UDP-GlcA/GalA, the contents of Met, Cys, GABA, Ser, Val, homoserine, 3PGA, and FBP increased, while those of citrate, fumarate, malate, and succinate decreased. Abbreviations are as follows: 2OG, 2-oxoglutarate; 3PGA, 3-phosphogrecerate; ADP-Glc, ADP-Glc; DHAP, dihydroxyacetone phosphate; FBP, fructose 1,6-bisphosphate; F6P, fructose 6-phosphate; GABA, γ-amino butyrate; gEC, γ-glutamylcysteine; G1P, Glc 1-phosphate; G3P, glycerol 3-phosphate; G6P, Glc 6-phosphate; PEP, phosphoenolpyruvate; Ru5P, ribulose 5-phosphate; RuBP, ribulose 1,5-bisphosphate; S7P, sedoheptulose 7-phosphate; UDP-Ara, UDP-L-arabinose; UDP-Glc/Gal, UDP-Glc and UDP-Gal; UDP-GlcA/GalA, UDP-GlcA and UDP-GalA; UDP-GlcNAc, UDP-*N*-acetyl-D-glucosamine.

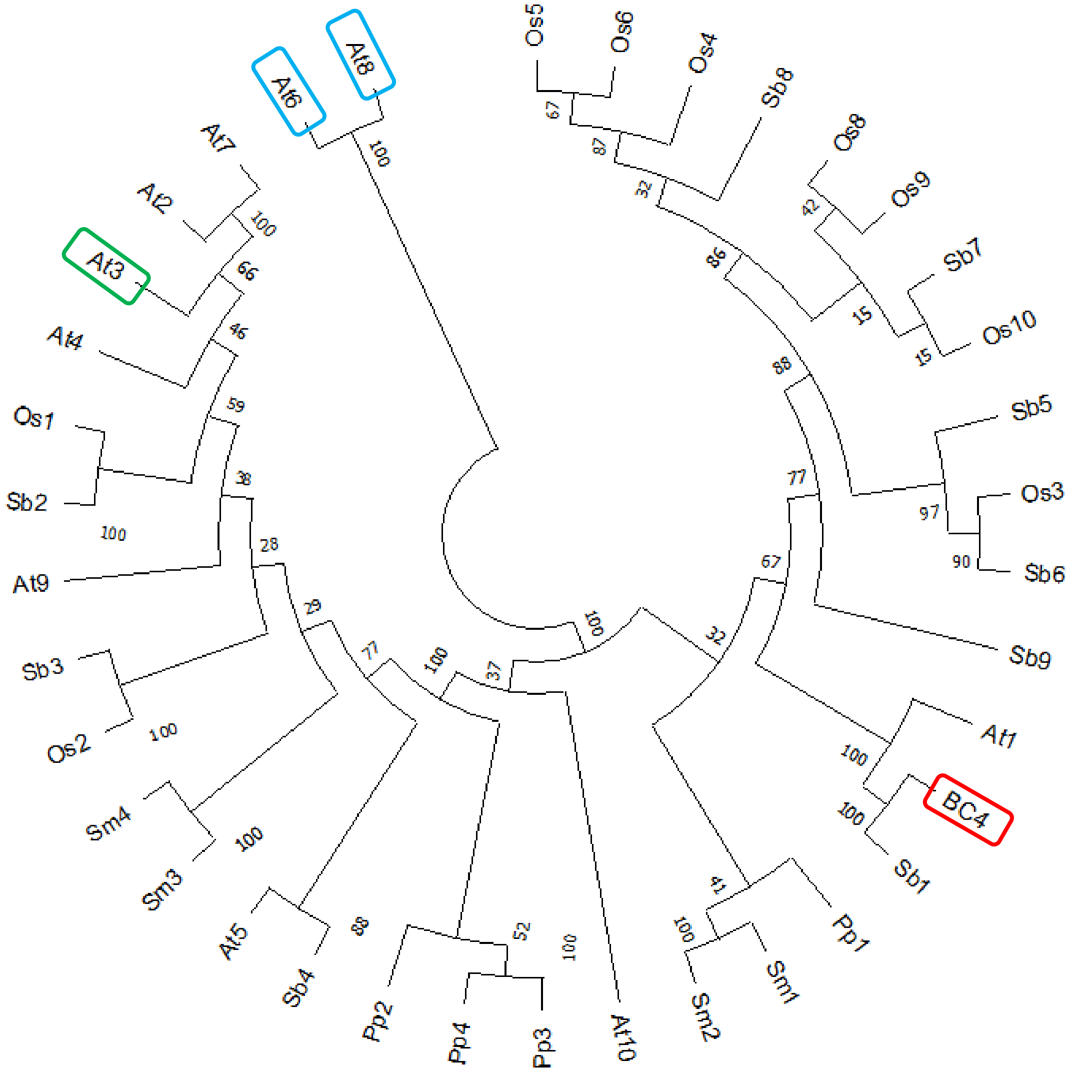


**Supplementary Figure 10. Phylogenetic tree of plant DUF1218 proteins.** The phylogenetic relationships of BC4 and other DUF1218 proteins were analyzed using MEGA software (version 11.0; Tamura et al., 2021). BC4, Arabidopsis proteins co-expressed with secondary cell wall CesA genes, and Arabidopsis MWL proteins were boxed with red, green, and blue boxes, respectively. The accession numbers and abbreviated names used are listed in Supplementary Table S3. At, Arabidopsis; Os, rice; Sb, *Sorghum bicolor*; Pp, *Physcomitrella patens*; Sm, *Selaginella moellendorffii*. Numbers at nodes represent bootstrap values.
