# Supplementary material for: The rice BRITTLE CULM 4 gene encodes a membrane protein affecting cellulose synthesis in the secondary cell wall
Source: Plant Cell Physiol. 2025 Aug 21;66(10):1444–53. doi: 10.1093/pcp/pcaf096 (PMC12540249; doi:10.1093/pcp/pcaf096)
Supplement: pcp-2024-e-00271-File008_pcaf096 [file pcp-2024-e-00271-file008_pcaf096.pdf]

Supplementary Table S1. Rice mutants with bc phenotype.

Mutant name	Causal gene	RAP number	Cell wall properties	References
bc1	COBRA-like 4	Os03g0416200	Reduced cellulose	Li et al. (2003)
bc3	DRP2B (dynamin)	Os02g0738900	Reduced cellulose	Hirano et al. (2010)
Bc6	CesA9 (cellulose synthesis)	Os09g0422500	Reduced cellulose and incereased xylan	Kotake et al. (2011)
bc7/Bc19	CesA4 (cellulose synthesis)	Os01g0750300	Reduced cellulose	Yan et al. (2007); Ma et al. (2021)
bc10	DUF266 membrane-protein	Os05g0170000	Reduced AGP and cellulose	Zhou et al.(2009)
bc12	Kinesin-4	Os09g0114500	Altered orientation of cellulose microfibril and increased lignin	Zhang et al. (2012)
bc15	membrane-associated chitinase-like protein	Os09g0494200	Reduced cellulose and increased xylan	Wu et al. (2012)
bc17	unknown protein	Os02g0450000	Reduced lignin	Li et al. (2022)
cef1	MYB103-like	Os08g0151300	Reduced cellulose and incereased xylan	Ye et al. (2015)
cef3	SCD2 (membrane-trafficking)	Os01g0928100	Reduced cellulose and xylan	Jiang et al. (2022)
fc18	UDP-GlcA decarboxylase	Os03g0278000	Reduced xylan and defective cellulose	Ruan et al, (2022)
fc19	IRX10 (xylan snthesis)	Os01g0926700	Reduced cellulose and xylan	Dang et al. (2023)
fc24	UDP-Glc epimerase	Os08g0374800	Reduced AGP and cellulose	Zhang et al. (2020)

Supplementary Table S2. DNA markers used for positional cloning

[a]Marker with amplified fragment with different length from co39.

[b]Cleaved amplified polymorphic sequence. The restriction enzyme used is shown in parentheses.

[c]Single nucleotide polymorphism.

Marker	Primer	Sequene	Length (bp)	Type
**2842-Indel**	Forward	5'-AGTAAATCCTGGATGGCTCG-3'	266/236	Indel[a]
	Reverse	5'-TTCATGGTCGCCATGGATCG-3'		
**1552-Indel**	Forward	5'-GGACGGAGGTATTACTTAGC-3'	200/229	Indel
	Reverse	5'-TGTCACTGCTAATTGCCGG-3'		
**1389-SalI**	Forward	5'-GATAGCTTCATTCACCCACC-3'	351	CAPS[b] (SalI)
	Reverse	5'-GTTGGTACCCACTATAGTCC-3'		
**1389-SNP**	Forward	5'-GGCCTGGTTTCATAGTACTC-3'	262	SNP[c]
	Reverse	5'-CACTGTTCACAGTACTTGCG-3'		
**2838-SNP**	Forward	5'-CACAGAACTGAATCACACTG-3'	241	SNP
	Reverse	5'-GTGTGTCCTTTAACGGTTAG-3'		
**2542-SNP**	Forward	5'-CTAGTCATGCACCAAGTTTC-3'	284	SNP
	Reverse	5'-CTAGAAGCTACCTGTATTGC-3'		
**0399-HindIII**	Forward	5'-GTTGGCGTCGTTGGAAGAGC-3'	163	CAPS (HindIII)
	Reverse	5'-GTTTGCTGTGTACTTGGATCG-3'		
**3510-Indel**	Forward	5'-CCACTACACTGATACACCCG-3'	207/243	Indel
	Reverse	5'-ATTATGCACTGCGTGCTTGG-3'		

Supplementary Table S3. DUF1218 proteins used in phylogenetic analysis.

Abbreviation	Accession number or gene locus	Source plants
BC4	Os06g0114700	*Oryza sativa*
Os1	Os07g0462200	*O. sativa*
Os2	Os04g0678200	*O. sativa*
Os3	Os05g0435300	*O. sativa*
Os4	Os05g0433500	*O. sativa*
Os5	Os05g0433100	*O. sativa*
Os6	Os05g0433600	*O. sativa*
Os7	Os05g0434200	*O. sativa*
Os8	Os05g0434600	*O. sativa*
Os9	Os05g0435100	*O. sativa*
Os10	Os05g0433400	*O. sativa*
At1	AT5G17210	*Arabidopsis thaliana*
At2	AT3G15480.1	*A. thaliana*
At3	AT4G27435.1	*A. thaliana*
At4	AT1G61065.1	*A. thaliana*
At5	AT1G68220.1	*A. thaliana*
At6	AT1G31720.1	*A. thaliana*
At7	AT1G52910.1	*A. thaliana*
At8	AT4G19370.1	*A. thaliana*
At9	AT1G13380.1	*A. thaliana*
At10	AT4G21310.1	*A. thaliana*
Sb1	Sb10g001220.1	*Sorghum bicolor*
Sb2	Sb02g011110.1	*S. bicolor*
Sb3	Sb06g032990.1	*S. bicolor*
Sb4	Sb07g009370.1	*S. bicolor*
Sb5	Sb09g021425.1	*S. bicolor*
Sb6	Sb09g021450.1	*S. bicolor*
Sb7	Sb09g021440.1	*S. bicolor*
Sb8	Sb09g021420.1	*S. bicolor*
Sb9	Sb01g026840.1	*S. bicolor*
Pp1	Pp053297	*Physcomitrella patens*
Pp2	Pp190444	*P. patens*
Pp3	Pp084743	*P. patens*
Pp4	Pp233170	*P. patens*
Sm1	Sm444273	*Selaginella moellendorffii*
Sm2	Sm446262	*S. moellendorffii*
Sm3	Sm154926	*S. moellendorffii*
Sm4	Sm266989	*S. moellendorffii*
